# Big and Free Fractions of Gamma-Glutamyltransferase: New Diagnostic Biomarkers for Malignant Mesothelioma?

**DOI:** 10.3390/diagnostics12020311

**Published:** 2022-01-26

**Authors:** Rudy Foddis, Maria Franzini, Alessandra Bonotti, Riccardo Marino, Roberto Silvestri, Poupak Fallahi, Dante Chiappino, Michele Emdin, Aldo Paolicchi, Alfonso Cristaudo

**Affiliations:** 1 Department of Translational Research and of New Technologies in Medicine and Surgery, University of Pisa, 56126 Pisa, Italy; maria.franzini@unipi.it (M.F.); riccardo.marino@med.unipi.it (R.M.); poupak.fallahi@unipi.it (P.F.); aldo.paolicchi@unipi.it (A.P.); 2 Preventive and Occupational Medicine, University Hospital of Pisa, 56126 Pisa, Italy; a.bonotti@yahoo.it (A.B.); r.silvestri17@gmail.com (R.S.); alfonso.cristaudo@unipi.it (A.C.); 3 Department of Radiology, Fondazione CNR Regione Toscana “G. Monasterio”, 54100 Massa, Italy; dante.chiappino@ftgm.it; 4 Cardiovascular Medicine Division, Fondazione Toscana “G. Monasterio”, 56124 Pisa, Italy; emdin@ftgm.it; 5 Institute of Life Sciences, Scuola Superiore Sant’Anna, 56127 Pisa, Italy

**Keywords:** mesothelioma, biomarkers, diagnosis, gamma-glutamyltransferase

## Abstract

Malignant pleural mesothelioma (MPM) is a cancer mainly caused by asbestos fiber inhalation, characterized by an extremely long latency and poor prognosis. Recently, researchers have focused on testing the diagnostic ability of several biomarkers. Gamma-Glutamyltransferase (GGT) has been demonstrated to be the sum of several GGT sub-fractions activity, classified based on their molecular weight in big-GGT, medium-GGT, small-GGT, and free-GGT. This work aims to evaluate whether specific GGT fractional enzymatic activity patterns could be helpful in MPM diagnosis. We analyzed blood samples from 175 workers previously exposed to asbestos, 157 non-exposed healthy subjects, and 37 MPM patients through a molecular exclusion chromatographic method. We found a specific profile of GGT fractions activity, significantly associated with MPM, resulting in an increase in b-, m- activity, along with an evident, yet not significant, decrease in f-activity. Receiver-operating characteristic (ROC) analysis showed that the best Area Under Curve (AUC) value resulted from the combined index b/f (0.679, 95% CI: 0.582–0.777). Combining the b-/f-GGT activity with the levels of serum mesothelin-related protein (SMRP; another promising MPM biomarker) improved the diagnostic accuracy, increasing the AUC value to 0.875 (95% CI: 0.807–0.943, *p* = <0.0001). Since MPM has a specific pattern of GGT enzymatic activity, we could hypothesize that GGT fractions play different specific biochemical roles. The improvement in the diagnostic power given by the combination of these two biomarkers confirms that the strategy of biomarkers combination might be a better approach for MPM diagnosis.

## 1. Introduction

Malignant pleural mesothelioma (MPM) is characterized by very long latency periods, sudden clinical onset, and extremely poor prognosis [1]. Identifying a panel of biomarkers to diagnose early grades is still the goal of many researchers [2,3]. While MPM is a rare cancer, its incidence is expected to increase dramatically due to the worldwide use of asbestos, the main etiological factor, over the past decades. Indeed, prolonged inhalation of asbestos fiber can form oxidized iron bodies, leading to the production of reactive oxygen species (ROS) and inducing reactive hyperplasia [4] that represents a primary step in mesothelioma development.

Gamma-Glutamyltransferase (GGT) is a type II membrane glycoprotein composed of a heavy and a light subunit linked by non-covalent bonds. GGT plays a crucial role in the translocation of amino acids across the plasma membrane. The activity of plasma GGT has been used for a long time as a liver function test and marker of alcohol abuse [5], but, more recently, increased GGT has been shown as a marker of oxidative stress [6], leading to cancer development and progression [7]. Accordingly, we showed that exposure to a subtoxic concentration of crocidolite asbestos triggered a GGT overexpression in THP-1 macrophagic cells, suggesting the possible involvement of GSH/GGT-dependent pro-oxidant reactions in the pathogenesis of MPM [8]. For this reason, GGT could be a diagnostic marker for MPM. Through the molecular exclusion chromatographic method, carried out on an FPLC system (Fast Protein Liquid Chromatography), it has been possible to identify and quantify four GGT fractions in human plasma, named according to their molecular weight: big-GGT (b-GGT), small-GGT (s-GGT), medium-GGT (m-GGT), and free-GGT (f-GGT) [9]. The fractional GGT method has improved the diagnostic use of GGT in the liver pathology field, the most traditional application of GGT. Indeed, b-GGT presents the best sensitivity and specificity for the diagnosis of steatosis, while s-GGT is more suited for chronic viral hepatitis [10]. These first results have raised the interest for GGT fractional enzymatic activity in other medical fields. For example, some in vitro studies showed that neoplastic epithelial cell lines other than the liver, including melanoma, prostate cancer, and bronchial epithelium, release GGT activity in the medium mainly as a b-GGT-fraction, explaining the increase in serum GGT observed in diseases of other organs [11].

Thus, regarding the role of cellular GGT in carcinogenesis [12] and plasma GGT as a risk factor for neoplastic-related mortality [13], this study aimed to identify a possible asymmetric distribution of GGT fractions among patients with MPM and people without neoplastic diseases. We also compared the diagnostic power of GGT fractions with serum mesothelin-related protein (SMRP), which is currently one of the best diagnostic biomarkers for MPM. Additionally, since we observed that the combination of SMRP and plasmatic osteopontin (pOPN) in a single biomarker resulted in increased accuracy [2], we compared the diagnostic power of this latter with that of GGT fractions as well.

## 2. Materials and Methods

### 2.1. Patients

This study was approved by the ethical committee for pharmaceutical experimentation at Pisa Hospital. All patients were recruited from January 2010 to December 2015 and gave written informed consent. Subjects with MPM (n = 37) were included in the study from consecutive patients presenting at the University Hospital of Pisa. The small number of MPM cases is due to the rarity of this cancer. Previously asbestos-exposed workers (pe-W, n = 175) were selected from a cohort of people followed up for cancer screening purposes at the University Hospital of Pisa. The 157 healthy non-exposed subjects (ne-HS, n = 157) were selected among those enrolled in the MEHLP Study (G. Monasterio Tuscany Foundation, Pisa, Italy). All MPM subjects were males. Both pe-W and ne-HS subjects included in the study were matched with MPMs for gender, age, and total GGT activity.

MPM patients were recruited at the time of diagnosis before beginning any treatment. All MPMs were epithelioid sub-type, being the more representative one between mesotheliomas, as histologically and/or cytologically confirmed. Mixed and sarcomatoid mesothelioma were excluded because of the paucity of available cases. Subjects with total GGT two-fold higher than the upper reference limit (110 U/l) and other hepatic transaminases out of the negative range were excluded to limit the bias deriving from possible misdiagnosed liver diseases. The pe-W subjects were recruited within a population of workers previously exposed to asbestos undergoing a preventive cancer program. These subjects underwent clinical examination, including chest radiography, functional respiratory tests (FRT), and, in some cases, low-dose computerized tomography. Based on the findings, the subjects were divided into the following groups: 70 (40%) benign respiratory diseases (BRD), 62 (35.4%) healthy-exposed subjects, and 43 (24.6%) patients with pleural plaques or unspecific lung nodules (<10 mm diameter). BRD included lung asbestosis (n. 2; 2.9%), emphysema (n. 5; 7.1%), chronic obstructive pulmonary disease (COPD; n. 5; 7.1%), silicosis (n. 8; 11.4%) and bronchiectasis (n. 5; 7.1%). All subjects previously exposed to asbestos were also characterized by their employment, occupational segment, and years of asbestos exposure. Since previous reports showed a more or less consistent association between increase/decrease in total GGT activity, or its fractions, and some physiologic or pathological conditions such as alcohol habits, diabetes, arterial blood hypertension, and other cardiovascular diseases, these parameters and their potential role as confounding factors were considered in the statistical analysis.

### 2.2. Biomarker Assays

Only for previously exposed to asbestos subjects and MPM cases, the serum-soluble mesothelin-related peptides (SMRP) concentration was measured using a sandwich-type Elisa, called Mesomark (Cisbio International, Gif/Yvette, France), and the plasmatic concentration of osteopontin (pOPN) was measured using the Osteopontin Assay Kit (IBL, Gunma, Japan) according to manufacturer’s instructions. Absorbance at 450 nm was used to quantify the SMRP/pOPN concentration in nmol/L by comparing the mean of the duplicate measurement with a calibration curve fitted by CourbesRD software (Installshield Corporation, Inc, France).

### 2.3. Fractional GGT Analysis

Analysis of total and fractional GGT was performed using plasma-EDTA samples (0.02 mL), as described previously [9], and an FPLC (fast protein liquid chromatography) system (AKTA purifier, GE Healthcare Europe, Milan, Italy) equipped with a gel filtration column (Superose 6 HR 10/300 GL, GE Healthcare Europe) and a fluorescence detector (Jasco FP-2020, Jasco Europe, Lecco, Italy). Separation of fractional GGT was obtained by gel filtration chromatography, and the enzymatic activity was quantified by post-column injection of the fluorescent substrate for GGT, gGluAMC. The enzymatic reaction, in the presence of gGluAMC 0.030 mmol/L and glycylglycine 4.5 mmol/L, proceeded for 4.5 min in a reaction coil (PFA, 2.6 mL) kept at the 37 °C in a water bath. The fluorescence detector operating at excitation/emission wavelengths of 380/440 nm detected the AMC signal; the intensity of the fluorescence signal was expressed in arbitrary fluorescence units (f.u.). Under these reaction conditions, the area under the curve is proportional to GGT activity. The total area, between 10 and 25 mL elution volume, and the fractional GGT area were calculated by a MatLab program (Version 7 MathWorks, Inc.) to resolve overlapping peaks; the curve fitting was conducted with a nonlinear least-squares minimization algorithm using four exponentially modified Gaussian (EMG) curves. The reaction was calibrated by analyzing plasma samples with known total GGT activity (standards); the slope of the calibration curve was used to convert the total and fractional GGT area into activity values expressed as U/L.

### 2.4. Statistical Analysis

GGT fraction, SMRP, and pOPN were explored to analyze their normality, using the Kolmogorov–Smirnov test. Since all variable distributions were not Gaussian, all values were shown as median, 25th and 75th percentiles, and the Mann–Whitney test was used to assess the differences between groups.

Logistic regression was used to determine the weight given to each marker and then to calculate a specific formula to provide a combined risk index. In order to estimate whether this marker combination might increase the performance of the markers in MPM detection, receiver-operating characteristic (ROC) curves were plotted, and the areas under curves (AUC) were calculated with their 95% confidence intervals (95% CI) using standard techniques to evaluate the sensitivity and specificity of each marker and their combination. The Youden index (1 + Sensitivity − (1 − Specificity)) was used to assess the best cut-off for each marker or marker combination. Statistical analysis was performed with SPSS v20.0 (Statistical Package for the Social Sciences).

## 3. Results

### 3.1. Patients’ Characteristics

The distribution of age, smoking habits, hepatic and cardiovascular diseases, and diabetes among ne-HS, pe-W, and a subset of 19 MPM (sMPM) is shown in Table 1. The same information was not available for the remaining 18 MPM subjects.

### 3.2. GGT Fractions Analysis

Each person in the study had a Total GGT value ranging from 6.51 to 106.58. The median and distribution values of the two non-neoplastic groups (ne-HS and pe-W) did not differ for any fractions except for the b-GGT and m-GGT, with the median of ne-HS being significantly lesser than the pe-W (Table 2).

The comparison between GGT fractions of those from both ne-HS and pe-W free from diabetes, hepatitis, and cardiovascular diseases did not influence this difference.

The sMPM group had a higher level of enzymatic activity of all the GGT fractions except for the f-fraction compared to the ne-HS. A significant difference was also observed between the sMPM group and the merged pe-W + ne-HS group concerning the b- and m-fractions (Table 2). When we included the whole MPM group (MPM, n = 37) in the analyses, we also observed a significant difference between the MPM and the pe-W group for the b- and m-fractions. Additionally, a significant difference emerged between the MPM group and the merged pe-W + ne-HS group for the s-fraction.

Interestingly, fractions b- and m- GGT showed a significant increasing trend from ne-HS to MPM groups (*p* = 0.001), while f-GGT showed a decreasing trend, even if not statistically significant (Table 3).

Since b- and m-GGT and f-GGT had different trends, we investigate whether a ratio of the single fractions had a better discriminating performance. We found that the only combination having a statistical advantage over the b-fraction was b/f-GGT fractions (Table 4).

**Figure 1:**
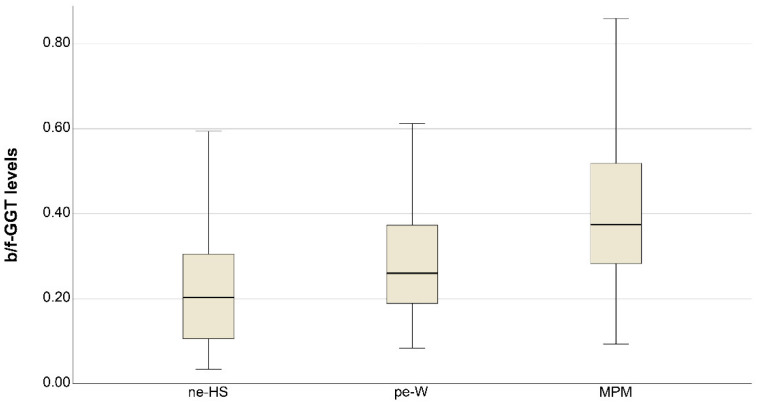
Graph showing the big/free-GGT ratio levels (b/f GGT) in the group of non-exposed subjects (ne-HS), previously exposed workers (pe-W), and patients affected by malignant mesothelioma (MPM). Box represents the first and the third quartile; the black line corresponds to the median value; the whiskers are 1st and 99th percentile.

The b/f-GGT ratio levels were statistically different between the three groups of studied subjects, as shown in Figure 1 (*p* < 0.0001).

### 3.3. SMRP vs. GGT Fractions Comparison

Median values for SMRP were significantly different between the pe-W and MPM patients (0.88 nM vs. 1.95 nM, respectively, *p* < 0.0001).

ROC curve analysis was performed for each GGT fraction, the b/f-GGT ratio, and SMRP. The resulting AUC values are shown in Table 4.

**Figure 2:**
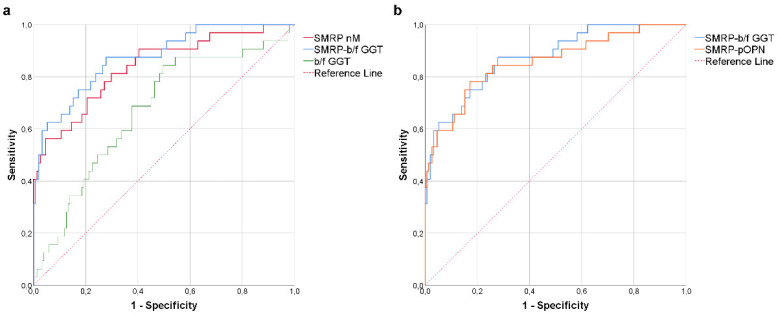
(**a**) ROC curve describing the diagnostic power of mesothelioma patients (MPM) vs. previously exposed workers (pe-W), by soluble mesothelin-related peptide (SMRP), big/free-GGT activity ratio (b/f GGT), and the combination of the two biomarkers (SMRP-b/f GGT); (**b**) comparison between the ROC curve of SMRPb/f GGT and combination of SMRP and plasmatic osteopontin (SMRP-pOPN).

Since SMRP and b/f-GGT had the better AUC values, they were combined in a single variable (SMRP-b/f GGT) using logistic regression. This combination improved the AUC to 0.875, as shown in Figure 2a (95% CI: 0.807–0.943, *p* = <0.0001). The best cut-off of the combined risk factors, resulting from the Youden index, was 0.10, associated with a combination of 87.50% sensitivity and 72.19% specificity. In a previous study, we reported that the combination of SMRP and pOPN in a single variable (SMRP-pOPN) significantly increased the diagnostic accuracy compared with the individual biomarkers alone [2]. Interestingly, when we compared the AUC of SMRP-pOPN with that of SMRP-b/f GGT, the latter was slightly greater (0.857, 95% CI: 0.776–0.937 vs. 0.875, 95% CI: 0.807–0.943) Figure 2b.

## 4. Discussion

Malignant mesothelioma is an asbestos-related cancer of the serosal membranes that can affect pleura, peritoneum, pericardium, and the tunica vaginalis testis, characterized by chemo- and radio-resistance. The worldwide use of asbestos in the last century and its high bio-persistence account for a large proportion of people exposed to asbestos for occupational or environmental reasons. Despite many countries banning the use and production of asbestos in the early 1990s, we are currently facing an increasing number of MPM cases due to past exposure. To date, however, no effective diagnostic tools exist for the surveillance of exposed individuals, and most MPMs are diagnosed at advanced stages. For this reason, many authors are evaluating the significance of biological indicators as biomarkers for the screening and early diagnosis of MPM.

Serum mesothelin-related protein (SMRP) is considered to be the most reliable marker [2,14].

SMRP by itself and other biomarkers currently under investigation do not possess an optimal combination of sensitivity and specificity. Nevertheless, some authors have demonstrated that the combination of different biomarkers can improve the diagnostic power [2,15,16].

Recently, numerous epidemiological studies have definitely ascertained that serum GGT elevation, even within the normal reference range, is associated with higher mortality of all causes, as well as with cardiovascular [17] and cancer-related mortality in the general population, independently from liver disease and alcohol abuse [13,18,19]. The association between serum GGT levels and the incidence of cancer (in general) and some site-specific cancer types was investigated in two large population-based cohort studies [18,19] and reviewed by Kunutsor et al. [13]. These studies reported a significant association between GGT levels and increased risk of developing digestive and respiratory/intra-thoracic malignancies in both genders. In all these previous studies, cancer risk correlated with total GGT. There is also consistent literature showing a correlation between total GGT amount and arterial blood hypertension, Diabetes Mellitus Type II (DMII) [20,21,22], cardiac [13,23], and hepatic diseases [5,24], but no data is yet available regarding any specific pattern of GGT fractional activity. In our study, a specific GGT fractional enzymatic activity has not been associated with any of the latter diseases. It must be outlined that all these subjects had total GGT values below two-fold the upper reference bound, limiting the possibility that our data are affected by the presence of subclinical hepatic alterations. Moreover, most people in the three groups had total GGT values below 55 U/l (290 subjects, 92.4%). However, we found that mesothelioma patients have a specific GGT fractional pattern, demonstrating that GGT-fractions may have a specific diagnostic meaning regardless of the total amount of GGT.

The most interesting result from the GGT fraction analysis is that the pattern associated with MPM differs not only from healthy subjects but also from those previously found in other diseases, such as muscular dystrophy [17], non-alcoholic fatty liver, and liver diseases in general [10,25]. These non-neoplastic patterns were characterized by an increase in total GGT and all single fractions, with s-GGT particularly specific for hepatic damage. In our study, MPMs showed a sensible increase in all GGT-fractions compared to the two combined control groups (ne-HS + pe-W), confirming previous observations. Additionally, MPMs were also characterized by a remarkable, though not statistically significant, decrease in the f-GGT fraction. We observed a positive trend in the b- and m-GGT fractional activity and a negative one for f-GGT, starting from ne-HS to pe-W and finally MPM. Since the physio- and pathologic meaning of GGT-fractions is still far from being well known, there is no chance for a sensible, comprehensive interpretation of our data. Nevertheless, only the b- and m-GGT among all fractions were significantly different between pe-W and ne-HS, and the b-GGT fraction is also the only one to be spontaneously released in the culture medium by GGT-expressing tumor cell lines or by activated inflammatory cells, as demonstrated in previous in vitro experiments [9,26,27]. Furthermore, the b-GGT fraction increase had been previously attributed to the inflammatory component of a heterogeneous group of diseases [10,26], and total GGT levels have been associated with established markers of inflammation [28,29]. Our pe-W group included several lung parenchymal diseases and mono-bilateral pleural plaques mostly due to asbestos. On this basis, we may speculate that the difference in b-GGT activity between ne-HS and pe-W could be attributed to an inflammatory thoracic condition, either clinical (those having pleural or parenchymal pathologies) or sub-clinical phlogosis, due to the presence of asbestos fibers potentially responsible for continuous inflammatory triggering or possibly to the sum of the two circumstances. The observation that the pe-W group free from pleural and parenchymal disease still had a median value of b-fraction activity higher than ne-HS (statically significant though with lesser strength) supports this theoretical explanation. Most likely, the physio-pathological role of any single GGT fraction is different, and this might explain the different up- or down-regulation observed in their activity according to different health conditions.

Due to the lack of specific symptomatology, MPM is frequently diagnosed at late stages when the chances of effective treatments are very poor. For this reason, several potential biomarkers of early diagnosis have been investigated. By itself, none of these biomarkers reached the levels of sensitivity and specificity required for screening purposes. Therefore, some authors have proposed using combined risk indexes deriving from the simultaneous application of different biomarkers. This approach has proved attractive to allow for an increase in diagnostic accuracy. To date, the combination of SMRP and plasma osteopontin (pOPN) provided the best diagnostic performance, with an AUC value of 0.873 ± 0.05 [2]. When we carried out a ROC curve analysis to establish the diagnostic accuracy of the combined SMRP + pOPN in the present cohort, we found an AUC of 0.857. Interestingly, the diagnostic accuracy of the combined risk index given by SMRP and b-/f-GGT ratio was slightly higher (0.875), with high sensitivity and specificity. While the accuracy of the combined SMRP-b/f GGT reported here is still too low to allow its employment in the clinical practice, these results suggest the eligibility of GGT fractions activity for a selected panel of early diagnosis biomarkers to be tested in future research. Furthermore, analyzing the GGT fractions on a larger sample could allow a better estimation of the real sensitivity and specificity of GGT as a biomarker for MPM.

In conclusion, this study demonstrated that the epithelioid subtype MPM is characterized by a specific pattern of GGT fractional activity, regardless of the total amount of GGT. The peculiarity of this enzymatic profile is given by a significant increase in the activity of the b-GGT and m-GGT fractions, along with a simultaneous decrease in the f-GGT fraction, which is constantly much more represented in healthy people. The b/f-GGT activity ratio resulted as the best performing parameter in ROC analysis among all fractions or rate combinations of them. The use of b/f-GGT in combination with SMRP further increases the diagnostic power. In our study, non-exposed healthy subjects and exposed people, regardless of any benign respiratory disease, showed different b-GGT fraction activity. A new targeted research design is necessary to assess the role of b-GGT fractional activity as a biomarker of asbestos exposure. Our data confirm the complexity of the physio-pathological role of what we simplistically refer to as GGT activity, suggesting, however, that further research is needed to exploit its potential in both clinical and preventive applications.

## 5. Conclusions

Up to date, the fractional GGTs analysis has been mostly explored in the perspective of implementing liver diseases diagnosis. The results of our study demonstrated that MPM is associated with a specific pattern of GGT enzyme activity, suggesting a potential utility in the diagnosis of MPM, as well as providing further evidence for the hypothesis that GGT fractions play several specific biochemical roles.

## Figures and Tables

**Table 1:** Distribution of some physio- pathological conditions in the study population of non-exposed subjects (ne-HS), previously exposed workers (pe-W), and a subset of malignant mesothelioma patients (sMPM) for which information about concomitant conditions was available.

	ne-HS (n = 157)	pe-W (n = 175)	sMPM (n = 19)
**Age**	66.22 (47–77)	65.46 (48–84)	69.16 (49–80)
**Hypertension**	44 (28.0%)	48 (27.4%)	5 (27.8%)
**Hepatic diseases**	0 (0.0%)	7 (4.0%)	2 (10.5%)
**Cardiovascular diseases**	36 (22.9%)	66 (37.7%)	7 (36.8%)
**Diabetes**	28 (17.8%)	20 (11.4%)	4 (21.0%)

**Table 2:** Summary of the analysis of GGT fractions distribution in non-exposed subjects (ne-HS), previously exposed workers (pe-W), and a subset of malignant mesothelioma patients (sMPM) for which information about concomitant conditions was available; data are expressed as median (25th–75th percentile) of enzymatic activity (U/L).

	ne-HS (n = 157)	pe-W (n = 175)	sMPM (n = 19)	pe-W vs. ne-HS (*p*-Value)	sMPM vs. ne-HS (*p*-Value)	sMPM vs. pe-W (*p*-Value)	sMPM vs. pe-W+ne-HS (*p*-Value)
**Tot-GGT**	22.91 (16.77–34.61)	24.27 (18.09–34.07)	24.29 (22.39–37.88)	ns	ns	ns	ns
**b-GGT**	2.46 (1.00–4.41)	2.93 (1.95–4.90)	3.95 (3.06–5.38)	0.004	0.002	ns	0.018
**m-GGT**	0.51 (0.22–0.98)	0.70 (0.37–1.22)	0.83 (0.53–1.42)	0.002	0.002	ns	0.029
**s-GGT**	7.28 (4.46–13.00)	8.88 (5.16–14.32)	10.00 (8.29–17.30)	ns	0.010	ns	ns
**f-GGT**	11.84 (9.39–15.70)	11.04 (9.14–13.90)	10.83 (8.79–13.50)	ns	ns	ns	ns

**Table 3:** Summary of the analysis of GGT fractions distribution when including the whole MPM group (MPM), the non-exposed subjects (ne-HS) and the previously exposed workers (pe-W); data are expressed as medians (25th–75th percentile) of enzymatic activity (U/L).

	ne-HS (n = 157)	pe-W (n = 175)	MPM (n = 37)	pe-W vs. ne-HS (*p*-Value)	MPM vs. ne-HS (*p*-Value)	MPM vs. pe-W (*p*-Value)	MPM vs. pe-W+ne-HS (*p*-Value)
**Tot-GGT**	22.91 (16.77–34.61)	24.27 (18.09–34.07)	26.28 (21.16–38.25)	ns	ns	ns	ns
**b-GGT**	2.46 (1.00–4.41)	2.93 (1.95–4.90)	4.06 (2.84–6.94)	0.004	0.001	0.020	0.001
**m-GGT**	0.51 (0.22–0.98)	0.70 (0.37–1.22)	0.77 (0.53–1.48)	0.002	0.001	0.043	0.002
**s-GGT**	7.28 (4.46–13.00)	8.88 (5.16–14.32)	9.78 (6.97–15.86)	ns	0.014	ns	0.046
**f-GGT**	11.84 (9.39–15.70)	11.04 (9.14–13.90)	11.06 (9.17–13.26)	ns	ns	ns	ns

**Table 4:** Area under the curve (AUC) values in order of increasing diagnostic power for the free-, small-, medium-, big-GGT fractions (f-, s-, m- and b-GGT), the total-GGT (Tot-GGT) and the ratio between big and free fractions (b/f-GGT) and big and medium fractions (b/m-GGT). The 95% CI indicates the 95% confidence interval of the AUC.

	AUC	95% CI	*p*-Value
**f-GGT**	0.477	0.377–0.577	ns
**Tot-GGT**	0.567	0.469–0.666	ns
**s-GGT**	0.574	0.476–0.671	ns
**m-GGT**	0.610	0.514–0.705	0.036
**b-GGT**	0.633	0.532–0.734	0.011
**b/f-GGT**	0.679	0.582–0.777	0.001
**b/m-GGT**	0.484	0.379–0.589	ns

## Data Availability

The data presented in this study are available on request from the corresponding author. The data are not publicly available due to ethical reasons.
